# Deciphering the molecular tapestry of schizophrenia: integrating transcriptomics, neuroimaging, and clinical data for precision medicine

**DOI:** 10.1038/s41398-025-03692-x

**Published:** 2025-11-21

**Authors:** Jia-Ni Zhao, Yun-Qi Wang, Min Liu, Bao-Bin Guo, You-Ren Wang, Bo Gao, Miao-Yan Liu, Wen-Jun Wu, Di Wu, Ya-Hong Zhang, Zhen Yuan, Li Zhu, Guo-Lin Ma, Long-Biao Cui, Yue-Lang Zhang

**Affiliations:** 1 Department of Radiology, The Second Affiliated Hospital of Xi’an Jiaotong University, Xi’an, China; 2 Schizophrenia Imaging Lab, Xijing 986 Hospital, Fourth Military Medical University, Xi’an, China; 3 Shaanxi Provincial Key Laboratory of Clinic Genetics, Fourth Military Medical University, Xi’an, China; 4 Department of Psychiatry, Xijing Hospital, Fourth Military Medical University, Xi’an, China; 5 Department of Psychiatry, Xi’an Gaoxin Hospital, Xi’an, China; 6 Centre for Cognitive and Brain Sciences, University of Macau, Macau SAR, China; 7 Faculty of Health Sciences, University of Macau, Macau SAR, China; 8 Department of Radiology, General Hospital of Ningxia Medical University, Yinchuan, China; 9 Department of Radiology, China-Japan Friendship Hospital, Beijing, China

**Keywords:** Schizophrenia, Clinical genetics

## Abstract

This study employed a multi-omics approach to investigate the molecular and functional underpinnings of schizophrenia by integrating blood transcriptomic profiles, neuroimaging-derived brain phenotypes, and clinical symptomatology. RNA sequencing of blood samples from 43 patients with schizophrenia and 60 healthy controls identified 994 differentially expressed genes (DEGs), the vast majority of which were downregulated (n = 921, |FC| > 1.5, P < 0.05), with enrichment in pathways related to neuronal development and inflammation. Concurrent neuroimaging analyses revealed altered functional activation in key brain regions, including the prefrontal and anterior cingulate cortices. A Partial Least Squares correlation analysis demonstrated significant cross-modal relationships among gene expression, neuroimaging patterns, and clinical presentation. Furthermore, we identified six genes—*GRK2*, *KLF3*, *TAOK2*, *ARFGAP45*, *AP1M1*, and *GPAT2*—that were shared across gene sets associated with both brain function and clinical symptoms, suggesting a common transcriptional basis for these features of schizophrenia. Collectively, these findings provide novel insights into the integrated molecular and functional changes in schizophrenia, highlighting the value of a comprehensive multi-omics strategy to decipher its pathophysiology and potentially inform improved diagnostic and therapeutic strategies.

## Introduction

Schizophrenia, an enigmatic disorder that afflicts millions worldwide, presents an intricate tapestry of psychiatric and cognitive symptoms [1, 2]. The quest to decipher its origins and mechanisms has long been a formidable challenge in neuroscience [3]. Despite a consensus that schizophrenia arises from a complex interplay of genetic susceptibility and environmental influences, substantial progress, the precise etiology and pathophysiology of schizophrenia remain shrouded in mystery, posing significant hurdles for the development of targeted interventions [4]. In this study, we adopt a multidimensional approach to investigate the convergence of transcriptomics, brain function, and clinical manifestations in schizophrenia.

The landscape of schizophrenia research has been dramatically transformed by the acknowledgment of its intricate polygenic nature. Genome-wide association studies (GWAS) have illuminated the presence of a multitude of risk alleles scattered across the genome, each contributing a cumulative effect to the overall disease susceptibility [5, 6]. This complex genetic architecture, underpinned by the collective influence of numerous genetic variants, many of which reside in non-coding regions, implies a pivotal regulatory function in gene expression. These regulatory elements may be sensitive to environmental stimuli, potentially triggering the cascade of events that culminate in the manifestation of schizophrenia [7]. Coinciding with these genetic insights, neuroimaging technologies have peeled back the layers of schizophrenia to expose an intricate interplay of cognitive-behavioral disturbances and pronounced abnormalities in brain architecture and functionality [8, 9]. Among the most compelling findings are the disruptions within the default mode network (DMN), a network inherently associated with self-referential thought and cognitive processes [10]. These DMN anomalies are intrinsically linked to the cognitive deficits that hallmark the disorder, providing a tantalizing link between brain structure and function and the clinical presentation of schizophrenia [11, 12]. Despite the invaluable contributions of clinical assessments, they often lack the biological resolution necessary to forge a definitive connection between the myriad symptoms of schizophrenia and their underlying pathophysiological substrates. This void underscores the imperative for a cohesive, multidisciplinary research paradigm that synthesizes transcriptional, neuroimaging, and clinical perspectives. The integration of these diverse domains is not merely an academic pursuit but a strategic necessity in the quest to identify robust biomarkers and viable therapeutic targets.

To address these gaps in knowledge, our study integrated transcriptomics, neuroimaging, and clinical data to provide a comprehensive understanding of the molecular and neurobiological underpinnings of schizophrenia. By intersecting the results from the transcriptome-neuroimaging and transcriptome-clinical analyses, we aim to pinpoint the most significant and potentially actionable genes. This approach is designed to uncover novel biomarkers and therapeutic targets, which could ultimately lead to improved diagnostic accuracy and more effective treatment strategies for this devastating condition.

## Methods

### Participants

**Fig. 1:**
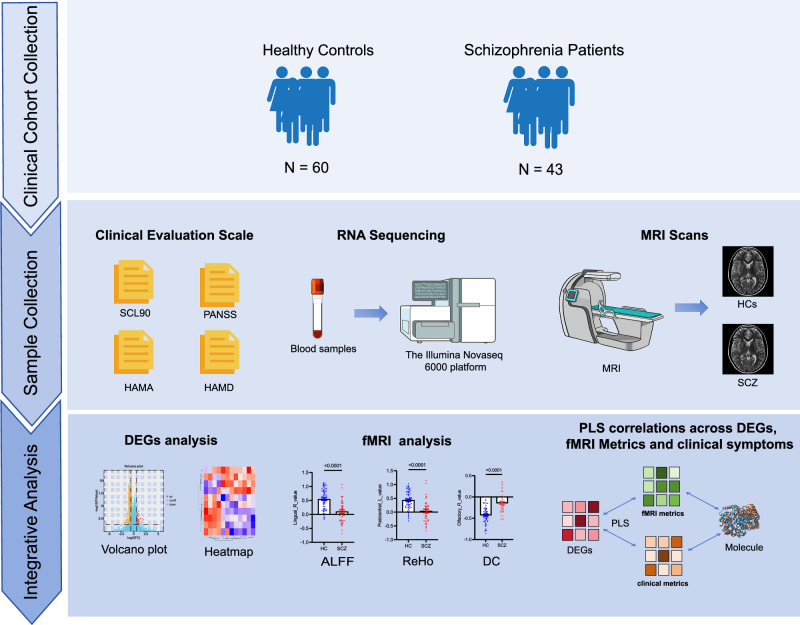
Overview of method. The figure outlines the study protocol involving the enrollment of 43 individuals with schizophrenia and 60 healthy controls (HCs). Blood samples were collected from all participants for RNA sequencing analysis. Concurrently, clinical assessments and magnetic resonance imaging (MRI) were conducted. Differential expression analysis was performed to identify variations in gene expression between the two groups, and functional MRI (fMRI) data were compared to highlight brain functional differences. Finally, partial least squares (PLS) analysis was employed to assess correlations among gene expression, clinical symptoms, and brain function, aiming to pinpoint genes potentially influencing the pathophysiological processes of schizophrenia.

A total of 43 schizophrenia patients and 60 age-, sex-matched healthy controls (HCs) were included in the present study (Fig. 1) [13]. Inclusion and exclusion criteria are described in our prior work [13]. Although no a priori power analysis was performed, our sample size aligns with previous studies in this field [13–16]. Post-hoc power calculations from our prior work with this cohort confirmed adequate statistical power [13]. Patients were recruited from the Department of Psychiatry, Xijing Hospital (First Affiliated Hospital of Fourth Military Medical University, Xi’an, China), and controls were recruited from local community. Individuals with schizophrenia were assessed using the Positive and Negative Syndrome Scale (PANSS), Symptom Checklist-90 (SCL-90), Hamilton Anxiety Scale (HAMA), and Hamilton Depression Scale (HAMD) assessing the clinical symptom severity [17]. For HCs, only cognitive tests were performed. This study was approved by the Institutional Ethics Committee, First Affiliated Hospital of Fourth Military Medical University. All participants provided written informed consent.

### RNA-seq data

#### Sample processing

Venous blood was collected from the antecubital fossa of each participant on the same day they underwent clinical assessments. The collection of blood samples did not necessitate a specific time or fasting requirements. A volume of 2.5 mL of blood was drawn into BD Vacutainer PAXgene RNA tubes, which were pre-filled with an RNA-stabilizing agent, and mixed and promptly stored at −80 °C.

#### Data acquisition

Subsequently, total RNA was isolated from the whole blood samples and processed within the same batch. RNA sequencing was carried out using the Illumina Novaseq 6000 platform. Poor-quality reads were removed using fastp (version 0.18.0) with: Q20 ≥ 90%, N bases ≤ 5%, and length ≥ 50 bp, and the remaining reads were aligned to the human reference genome hg19 with HISAT2 (version 2.2.4). The aligned reads were then assembled using StringTie (version 1.3.1) [13].

#### Differential expression analysis

The calculation of gene expression levels in the samples was carried out using the fragments per kilobase of transcript per million mapped reads (FPKM) method. For the analysis of differential RNA expression, we utilized the DESeq2 software, which models data based on raw read counts. Differentially expressed genes (DEGs) were identified using DESeq2 with FDR-adjusted *q* < 0.05 (Benjamini-Hochberg) and |log2FC | ≥ 1.5. Visualization of volcano plot, heatmaps, radar plot, and bubble chart was facilitated by the OmicShare tools, an online data analysis platform available for free at https://www.omicshare.com/tools.

### Image data

#### Image acquisition

The blood sample acquisition and the MRI scanning were performed on the same day. Subject underwent scanning using a General Electric (GE) Discovery MR750 3.0 T scanner with a standard 8-channel head coil. Scanning parameters are described in Supplementary Table 1. A custom-built head coil cushion and earplugs were used to minimize head motion and dampen scanner noise. During data acquisition, subjects were asked to remain alert with eyes closed and keep their head still.

#### Data preprocessing

Data were preprocessed using Data Processing Assistant for Resting State fMRI Advanced Edition DPABI (Version 8.2, available at http://rfmri.org/DPARSF) [18]. For each subject, the preprocessing steps included slice-timing correction, realigned to correct for head motion (exclusion criteria: ≤2.5 mm translation and/or ≤2.5°rotation), smoothing (FWHM = 6 mm, only for the ALFF analysis), and band-pass filtering (0.01–0.1 Hz, only for the ReHo and DC analysis).

#### ALFF, ReHo and DC calculation

Using the preprocessed resting state fMRI data, we calculated the ALFF, ReHo, and DC values for each subject. For ALFF, the data were filtered using a bandpass filter (0.01–0.08 Hz). Then, the ALFF values for all brain voxels were normalized. The ReHo value, also called the Kendall’s coefficient of concordance (KCC), of the time series of a given voxel with its nearest neighbors (27 voxels were considered) was calculated, similar to the standardization procedure for ALFF analysis, the whole-brain ReHo values are also standardized in DPABI Statistical analysis. For DC, the BOLD time process was extracted and associated with every voxel in the brain, and calculated the correlation of the time series for each voxel in the brain. We computed the DC as the sum of functional connections (binarized) for each voxel, and *Z* transformed the data to facilitate statistical analyses. A threshold of *r* > 0.25 was established for Pearson’s correlation coefficient. Lastly, for the ReHo and DC maps, smoothing (FWHM = 6 mm) was performed.

#### Statistical analysis

Statistical comparisons of ALFF, ReHo, and DC between patient and HC groups were conducted using two-sample t-tests. The use of this parametric test was justified because the data for all three metrics were confirmed to approximate a normal distribution (via the Shapiro-Wilk test) and exhibited homogeneity of variance between groups (via Levene’s test). Within-group variation is presented in the results as mean ± standard deviation. Following FDR correction, cluster-level *P* < 0.05 and voxel-wise *P* < 0.001 were considered statistically significant.

A Multivariate Partial Least Squares (PLS) correlation analysis was conducted to investigate the relationships between the gene expression levels of DEGs and phenotypes derived from imaging data or clinical symptoms. PLS is a popular method for identifying latent components by maximizing the covariance between two datasets [19, 20]. The PLS analysis was carried out utilizing the myPLS software package, available at https://github.com/danizoeller/myPLS [21].

## Results

### DEGs analysis in blood samples in schizophrenia patients

**Fig. 2:**
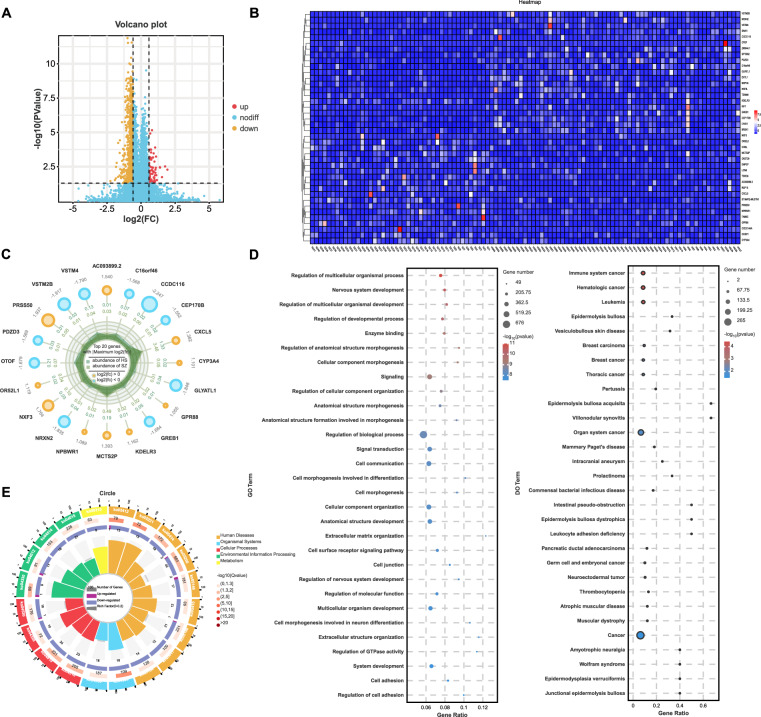
RNA-seq analysis between schizophrenia patients and healthy controls. **A** Volcano plot of differential expression genes (DEGs) in schizophrenia (schizophrenia vs HCs). **B** Heatmap illustrates the top 20 upregulated and downregulated genes. **C** Radar plot summarizes the top 10 changed genes across all samples. **D,** **E** Gene Ontology (GO), Disease Ontology (DO), and Kyoto Encyclopedia of Genes and Genomes (KEGG) analyses of the DEGs.

The baseline characteristics of the participants are presented in Supplementary Table 2. In accordance with previous studies, we observed DEGs in patients with schizophrenia (n = 994 at |FC| >1.5 and *P* value <0.05), with a total of 73 genes showing significant upregulation and 921 genes showing significant downregulation (Fig. 2A). The heatmap in Fig. 2B further elucidates the expression patterns of the top 20 upregulated and downregulated genes, revealing distinct clusters and expression levels between two groups. The radar plot in Fig. 2C provides a comprehensive view of the expression patterns across samples, underscoring the variability and consistency of gene expression changes in schizophrenia.

To gain insight into the biological classification of the identified DEGs, we conducted GO, DO, and KEGG (Fig. 2D and F) pathway enrichment analyses. The results suggest that these DEGs are involved in critical biological processes and pathways, while the KEGG enrichment circle plot in Fig. 2E indicates significant enrichment in pathways such as neuronal development, which are known to be relevant to the pathogenesis of schizophrenia.

### Distinct brain region activity differences in resting-state fMRI between schizophrenia patients and controls

**Fig. 3:**
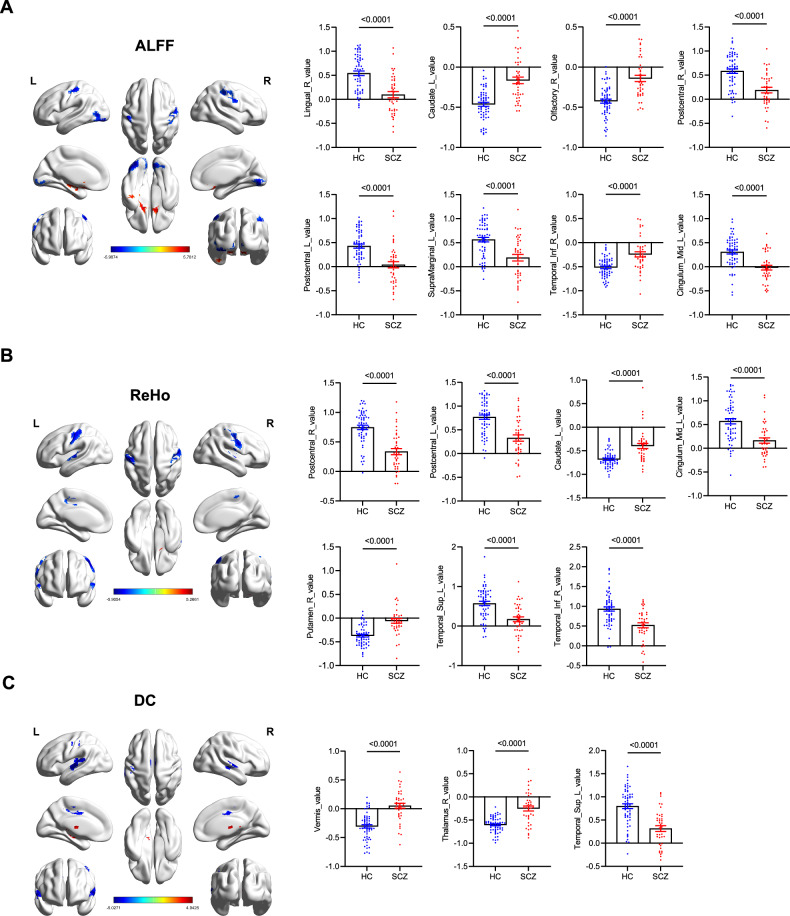
Functional magnetic resonance imaging (fMRI) analysis in schizophrenia patients and healthy controls. **A–C** Amplitude of low-frequency fluctuations (ALFF), regional homogeneity (ReHo), and degree centrality (DC) analysis between schizophrenia patients (n = 40) and healthy controls (HCs, n = 60). Statistical significance was determined by Student *t* test. *P < 0.05.

In our fMRI investigation, we discerned marked variations in neural activity patterns between schizophrenia and HCs. ALFF analysis indicated significant alterations, with patients exhibiting diminished activity in the right lingual gyrus, postcentral gyrus, left supramarginal gyrus, and left middle cingulum, yet heightened activity in the left caudate nucleus, right olfactory cortex, and right inferior temporal lobe (*P* < 0.0001; Fig. 3A). ReHo comparisons revealed significant modulations, particularly decreased coordination in the postcentral gyrus, left middle cingulum, left superior temporal lobe, and right inferior temporal lobe (*P* < 0.0001; Fig. 3A). DC analysis further exposed enhanced network centrality in the right thalamus and vermis, and juxtaposed with reduced centrality in the left superior temporal lobe among patients (*P* < 0.0001; Fig. 3C), pointing to disrupted neural connectivity in schizophrenia.

### Gene expression correlates with resting-state fMRI metrics and clinical symptoms

**Fig. 4:**
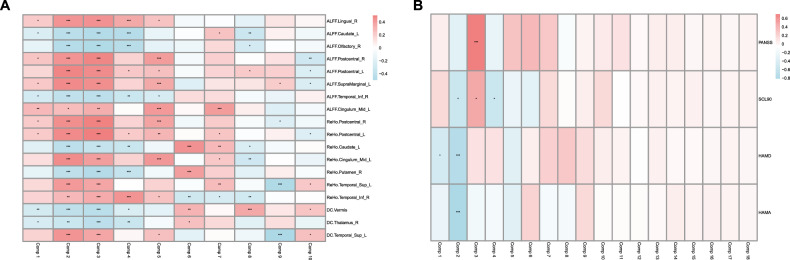
Correlation analysis between gene expression, neuroimaging, and clinical assessments in schizophrenia. **A**, **B** Heatmap depicts the association between differentially expressed genes (DEGs), functional magnetic resonance imaging (fMRI) (A) and clinical indices (B).

In the tertiary segment of our empirical findings, we performed a comprehensive correlation analysis to elucidate the inter relationships among DEGs, the 18 fMRI measurements that exhibited significant intergroup variance, and the clinical indices for schizophrenia. The results indicated a robust association between the transcriptomic profiles and the neuroimaging indices (Fig. 4A), as well as a significant linkage with the schizophrenia clinical evaluation scores (Fig. 4B). These observations suggest a pivotal connection between the transcriptome signatures in peripheral blood and the neural substrates of cognitive and affective dysfunction observed in schizophrenia, underscoring the integrative nature of transcriptome and neuroimaging biomarkers in the disorder.

### Correlation analysis between top genes and fMRI metrics or clinical symptoms

**Fig. 5:**
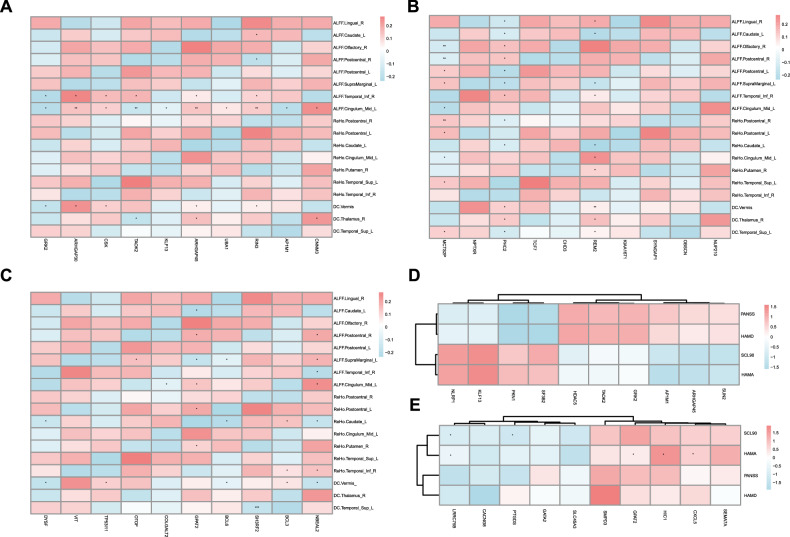
Correlation analysis of genes associated with fMRI metrics and clinical assessments. **A**–**E** Heatmaps illustrating the correlations between the top ten genes from Principal Components 1–3 and fMRI metrics (**A**-**C**) or clinical assessments (**D** and **E**).

In the subsequent analyses, Fig. 5A–E depict heatmaps and a Venn diagram elucidating the correlations and overlap between the top ten genes from Principal Components 1–3 and both fMRI metrics and clinical assessment scores. This reveals a common transcriptional basis influencing neuroimaging and clinical features of schizophrenia.

**Fig. 6:**
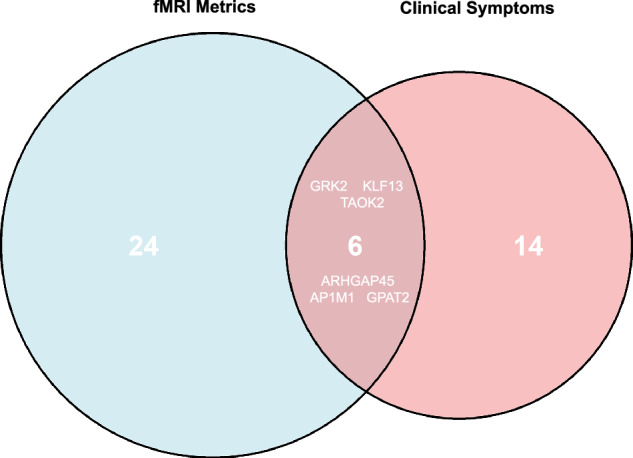
Intersection analysis of genes common to brain function and clinical symptomatology. A venn diagram illustrating six genes that are common to both brain function and clinical symptomatology-related gene sets.

Key genes, including *MCTS2P*, *PHC2*, *REM2*, *GPAT2*, and *NBEAL2*, exhibited significant associations with fMRI metrics. Correlation analysis with clinical symptomatology identified *LRRC75B* and *PTGDS* as negatively associated with SCL90, and *GPAT2*, *HIC1*, and *CXCL5* as positively correlated with HAMA scores. An intersection analysis revealed six genes-*GRK2*, *KLF3*, *TAOK2*, *ARFGAP45*, *AP1M1*, and *GPAT2*-common to both brain function and clinical symptomatology gene sets, highlighting a shared genetic etiology in schizophrenia’s neuroimaging and clinical manifestations (Fig. 6).

## Discussion

We combine data of a group of schizophrenia and HCs across three scales, including blood transcriptome, functional neuroimaging-derived brain phenotypes, and clinical symptoms, showing cross-scale associations that are key for understanding the biological mechanisms of schizophrenia. Integrating multiple abnormalities in brain function, pairwise interaction between MRI and transcriptomics have provided a way to establish the transcriptomic correlates of cerebral alterations in relation to schizophrenia [22]. Our study suggests that blood-sample gene expressions might provide an informative way to study participant-level transcriptome-neuroimaging associations in schizophrenia.

Our reported relationship between DEGs in blood samples and brain functional alterations in schizophrenia implies blood transcriptomic data to be an informative source to study transcriptome-neuroimaging associations in schizophrenia. Genetic association between schizophrenia and cerebral MRI has been demonstrated by means of independent study data (Psychiatric Genomics Consortium, CLOZUK, and UK Biobank) [23]. However, one of the obstacles in identifying transcriptome-neuroimaging associations of the disorder is that transcriptome and MRI data are commonly derived from different sources. Our previous study thus attempted to address this issue by collecting cross-scale data from one cohort of schizophrenia [22], and our observed correspondence between blood DEGs and brain DEGs is in line with the correlation of genetic effects between human blood and brain [24]. Our results of associations between blood DEGs and schizophrenia GWAS further suggests blood DEGs were significantly regulated by common genomic variants in schizophrenia, regardless of the examined ethnics [22]. Therefore, examinations of the participant-level associations between blood gene expression and imaging-derived brain phenotypes in schizophrenia might provide evidence of the disease-involved biological pathway that advances our understanding of the disease mechanisms.

Our RNA-seq analysis of blood from individuals with schizophrenia and demographically matched HCs has revealed a striking imbalance in gene expression, with 73 genes upregulated compared to 921 genes downregulated in schizophrenia. This preponderance of downregulated genes echoes a recent report [25], pointing to several possible etiological factors. Primarily, schizophrenia may involve dysregulation of transcriptional networks, culminating in the suppression of select gene cohorts [22, 26, 27]. Additionally, the chronic inflammatory and oxidative stress states observed in these patients likely contribute to the altered gene expression patterns, a hypothesis supported by our pathway enrichment analyses [28]. Furthermore, neuropsychiatric conditions such as schizophrenia are frequently associated with dysfunctional neuronal circuits. Aberrations in neurotransmitter systems, for example, dopamine and glutamate, may underlie reduced neuronal activity and subsequent downregulation of genes essential for neuronal integrity, synaptic plasticity, and communication. This is corroborated by our findings of a suppressed state in functional brain connectivity among schizophrenia patients, indicative of neuronal injury, neurodegenerative processes, and transcriptional imbalance. These observations align with recent pivotal studies that underscore the complex interplay between gene expression perturbations, neuroinflammation, and neurodegeneration in the pathophysiology of schizophrenia.

These biological underpinnings are compatible with polygenic contribution to the brain disorder of schizophrenia patients in vivo, involving neurochemical disturbance and neurodevelopment [29]. A recent meta-analysis identified brain structure in the cognitive networks, amygdala, hippocampus, and cerebellum typically showing associations with conceptually related cognitive domains in schizophrenia [30]. Meanwhile, declined cognitive ability is significantly associated with schizophrenia polygenic risk scores [31]. Cognitive dysfunction of schizophrenia is related to the altered whole blood gene expression of immunological process [32].

Cross-scale associations between gene expression and brain function were presented, showing that lower expression of DEGs in schizophrenia was associated with the reduced functional activity in the dorsolateral prefrontal cortex and anterior cingulate cortex. These regions have been broadly reported in previous neuroimaging studies in schizophrenia, where decreased neural activity and connectivity were observed in schizophrenia patients compared to healthy controls, underscoring the convergence of genetic and neuroimaging findings in the disorder. Within these regions (e.g., the anterior cingulate cortex and superior temporal cortex), downregulated expressions of immune-associated genes have been also demonstrated in schizophrenia [33]. Moreover, our reported transcriptome-neuroimaging associations at the participant level corroborate recent findings linking transcriptome and brain on the basis of brain spatial variations [34, 35], for instance, genes related to functions in the central nervous system development show overlapped pattern with brain volume changes in schizophrenia [36]. Cortical thickness alterations are in relation to heterogeneity between individuals with schizophrenia, with higher polygenic risk for schizophrenia associated with a greater number of regions with infra-normal deviations in cortical thickness [37]. Such associations between transcription and brain volume might be related to the crucial regulatory role of schizophrenia risk genes in human brain development [38]. In line with this, neuron- and astrocyte/oligodendrocyte progenitors-linked genes were identified based on the transcriptomic and polygenic manifestations of cortical thickness heterogeneity in schizophrenia [39]. Our observed participant-level relationship between gene transcription and brain function in schizophrenia show a great potential of integrating multi-omics features in future works on predictive models in schizophrenia.

Similarly, we showed that the white matter connectivity strength of brain hubs, such as superior frontal and superior parietal regions, is associated with blood gene expression of DEGs in schizophrenia [22]. This finding is in line with previous studies showing brain hubs to show higher heritability compared to the rest of the brain regions and to be related to a tight coupling of transcriptional profiles [40]. Schizophrenia risk genes specifically showed a brain transcriptional profile overlapped with the spatial pattern of disruptions in white matter connectivity [41]. Such an association between schizophrenia gene expression and brain dysconnectivity might reflect the genetic origins of widespread connectivity disruptions observed in schizophrenia, in particular for hubs and the rich club [42].

To elucidate genes concurrently associated with both genetic markers and clinical symptoms of schizophrenia, we intersected the two gene sets, identifying six overlapping genes. Notably, *GPAT2*, glycerol-3-phosphate acyltransferase 2, exhibited significant correlations with both sets of indicators. *GPAT2* is a member of the *GPAT* family involved in the biosynthesis of triglycerides and phospholipids, predominantly active in the endoplasmic reticulum and mitochondrial outer membrane [43]. While no prior studies have linked *GPAT2* to schizophrenia, its role in generating phosphatidic acid—a precursor to membrane phospholipids essential for cell membrane structure, function, and inter-neuronal interactions—is intriguing. The downregulation of *GPAT2* in schizophrenia suggests neuronal/glial damage and lipid metabolism dysregulation, aligning with schizophrenia pathophysiology. Consequently, *GPAT2* may serve as a potential biomarker for aiding in the diagnosis of schizophrenia.

Recent advances in functional neuroimaging have demonstrated how intrinsic connectivity networks can reveal the systems-level mechanisms underlying schizophrenia [44, 45]. Our findings using multiple rs-fMRI metrics align with this perspective - the observed ALFF abnormalities in prefrontal regions correspond to emerging evidence of default mode network dysregulation in schizophrenia [44], while the ReHo disruptions in sensory-motor areas may reflect local connectivity deficits associated with neuroinflammatory processes [46]. Furthermore, the DC alterations we identified in thalamocortical hubs resonate with studies linking these network properties to both genetic risk factors [45] and clinical symptom severity [47]. Together, these converging lines of evidence strengthen our integrated approach combining transcriptomic and neuroimaging data to map schizophrenia’s complex pathophysiology.

## Limitations

Our study has several limitations. Firstly, the use of blood samples reflects transcriptomic changes in circulation but not directly in brain tissue. Lastly, our study demonstrates correlations but does not establish causality; further experimental work is needed to clarify the causal pathways involved in schizophrenia.

## Conclusion

In summary, we found that blood-sample DEGs was a direct way to study individual variations in gene expression, and we also found that individual variations in gene expression showed an association with variations in brain function in schizophrenia. In other words, our research supports the disrupted brain function in patients with schizophrenia from the perspective of gene expression, which could be useful for understanding schizophrenia and searching for new targeted therapies. Given these findings, establishing transcriptomic correlates of brain function holds promise for building biomarkers that could contribute to the diagnosis and assessment of schizophrenia.

## Supplementary information

Supplementary materials

## Data Availability

The raw MRI data are available from the corresponding author L.-B.C. (lbcui@fmmu.edu.cn) upon reasonable request. The raw RNA-seq data are available from the corresponding author L.-B.C. (lbcui@fmmu.edu.cn) upon reasonable request after having all relevant approvals from China’s Ministry of Science and Technology related to the export of genetic information and materials relevant to this work.
