# Supplementary material for: Deciphering the molecular tapestry of schizophrenia: integrating transcriptomics, neuroimaging, and clinical data for precision medicine
Source: Transl Psychiatry. 2025 Nov 21;15:489. doi: 10.1038/s41398-025-03692-x (PMC12638306; doi:10.1038/s41398-025-03692-x)
Supplement: Supplementary file 1 — Supplementary materials [file 41398_2025_3692_MOESM1_ESM.docx]

**Supplementary Table 1.** Detailed Scanning Parameters.

|  | T1-weighted imaging | Functional imaging |
| --- | --- | --- |
| TR (ms) | 8.2 | 2000 |
| TE (ms) | 3.2 | 30 |
| Flip angle (◦) | 12 | 90 |
| Field of view (mm2) | 256 × 256 | 240 × 240 |
| Matrix | 256 × 256 | 64 × 64 |
| Slice thickness (mm) | 1 | 3.5 |
| Slice gap (mm) | 0 | 0 |
| Number of slices | 196 | 45 |

NA, not applicable; TE, echo time; TR, repetion time.

**Supplementary Table 2.** Demographical and Clinical Data of Participants.

| **Characteristic** | **Patients (n = 43)** | **HCs (n = 60)** | **Two-sided P value** |
| --- | --- | --- | --- |
| **Sociodemographic** |  |  |  |
| Age, y | 26.5 ± 9.4 | 26.1 ± 5.5 | .783^a^ |
| Sex, male/female | 23/20 | 36/24 | .510^b^ |
| Ethnicity, Han/others* | 43/0 | 60/0 |  |
| Education level, y | 12.7 ± 2.9 | 14.1 ± 2.9 | .078^c^ |
| **Clinical assessment^d^** |  |  |  |
| PANSS total score | 61.7 ± 17.4 | NA |  |
| PANSS positive score | 13.6 ± 6.5 | NA |  |
| PANSS negative score | 14.8 ± 8.3 | NA |  |
| PANSS general psychopathology score | 33.3 ± 8.3 | NA |  |
| **Intelligence^c,e^** |  |  |  |
| Information | 9.5 ± 3.1 | 12.7 ± 3.2 | 2.381 × 10^−6^ |
| Digit span | 11.5 ± 2.6 | 14.1 ± 3.2 | 8.905 × 10^−6^ |
| Digit symbol coding | 11.0 ± 2.7 | 15.6 ± 3.0 | 7.090 × 10^−11^ |
| Vocabulary | 10.8 ± 2.6 | 13.6 ± 3.0 | 1.088 × 10−6 |
| Picture completion | 8.3 ± 2.8 | 11.1 ± 3.0 | 2.146 × 10−6 |
| Block design | 10.6 ± 3.3 | 13.5 ± 2.4 | 1.956 × 10−6 |
| IQ | 97.6 ± 14.6 | 114.7 ± 17.5 | 7.312 × 10−7 |

HCs, healthy controls; NA, not applicable; PANSS, Positive and Negative Syndrome Scale; IQ, Intelligence quotient.

^a^Two sample t-test.

^b^χ^2^ test.

^c^Wilcoxon Rank Sum test.

^d^Seven patients had missing PANSS information.

^e^One healthy control did not take the intelligence test. Information and vocabulary were unavailable for one healthy control.

*Ya-Hong Zhang, Yu-Fei Fu, Xiao-Sa Li, Wen-Jun Wu, Di Wu, Long-Biao Cui identified participant ethnicity according to electronic health record.
